# Supplementary material for: Amoeba plate test with Acanthamoeba castellanii as an innovative tool for Nocardia recovery from sputum samples: a proof-of-concept study
Source: Microbiol Spectr. 2024 Nov 22;13(1):e01416-24. doi: 10.1128/spectrum.01416-24 (PMC11705944; doi:10.1128/spectrum.01416-24)
Supplement: Fig. S1 — APT assay overview. [file spectrum.01416-24-s0001.pdf]

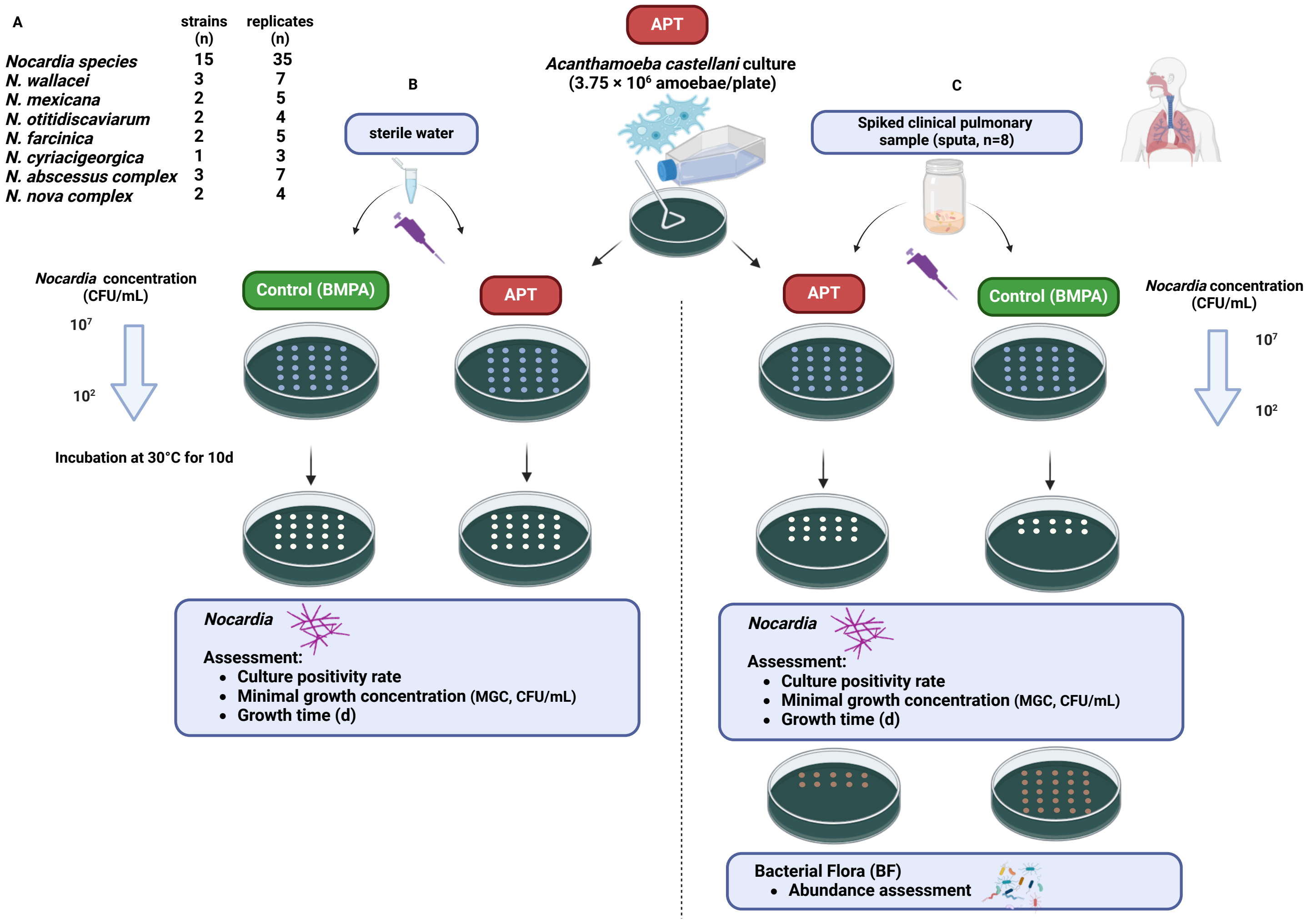


**Figure S1. APT assays overview.** (A) *Nocardia* species tested in the study, number of tested strains per species and total number of replicates. (B) *Nocardia* dilutions and APT procedure in sterile water. (C) *Nocardia* dilutions and APT procedure in clinical pulmonary samples (sputa)
