# Supplementary material for: Amoeba plate test with Acanthamoeba castellanii as an innovative tool for Nocardia recovery from sputum samples: a proof-of-concept study
Source: Microbiol Spectr. 2024 Nov 22;13(1):e01416-24. doi: 10.1128/spectrum.01416-24 (PMC11705944; doi:10.1128/spectrum.01416-24)
Supplement: Table S1 — APT and control MGC and growth times for Nocardia in sterile water. [file spectrum.01416-24-s0002.pdf]

Table S1. APT and control MGC and growth times for Nocardia in sterile water.

Nocardia species	Strain	Replicate number	MGC (CFU/ml)		Growth time	
			APT	Control	APT	Control
***N. wallacei* (n=7)**	EML 1472	1	10^3	10^3	2	2
		2	10^3	10^3	2	1
	EML 1473	1	10^2	10^4	3	2
		2	10^4	10^4	4	3
	EML 1474	1	10^2	10^3	3	2
		2	10^3	10^4	2	2
		3	10^4	10^3	2	2
***N. mexicana* (n=5)**	EML 1475	1	10^3	10^3	1	1
		2	10^3	10^5	2	2
	EML 1476	1	10^3	10^3	2	1
		2	10^3	10^3	2	2
		3	10^4	10^3	2	2
***N. otitidiscaviarum* (n=4)**	EML 1477	1	10^3	10^4	2	2
		2	10^3	10^3	1	1
	DSM 43242^T	1	10^3	10^3	2	1
		2	10^3	10^3	1	1
***N. farcinica* (n=5)**	EML 1478	1	10^2	10^4	3	2
		2	10^3	10^3	2	2
	EML 1479	1	10^2	10^2	4	3
		2	10^6	10^5	5	4
		3	10^3	10^4	3	2
***N. cyriacigeorgica* (n=3)**	EML 1480	1	10^2	10^3	7	3
		2	10^3	10^4	2	2
		3	10^3	10^3	2	2
***N. abscessus complex* (n=7)**	DSM 44432^T	1	$>10^7$	10^4	>10	4
		2	10^4	10^5	4	4
	EML 1481	1	10^6	10^5	7	7
		2	10^4	10^5	2	2
		3	$>10^7$	10^5	>10	5
	EML 1482	1	$>10^7$	$>10^7$	>10	>10
		2	$>10^7$	$>10^7$	>10	>10
***N. nova complex* (n=4)**	EML 1483	1	10^4	10^5	7	3
		2	$>10^7$	10^4	>10	4
		3	$>10^7$	10^3	5	3
	DSM 44481^T	1	10^3	10^3	2	4

CFU: Colony Forming Unit. APT: Amoebae Plate Test. MGC: Minimal growth concentration. EML: *Environnement Microbiologie Lyon* collection. DSM: *Deutsche Sammlung von Mikroorganismen collection*
