# Supplementary material for: Amoeba plate test with Acanthamoeba castellanii as an innovative tool for Nocardia recovery from sputum samples: a proof-of-concept study
Source: Microbiol Spectr. 2024 Nov 22;13(1):e01416-24. doi: 10.1128/spectrum.01416-24 (PMC11705944; doi:10.1128/spectrum.01416-24)
Supplement: Table S2 — APT and control MGC and growth times for Nocardia, type, and abundances of BF overgrowth in clinical sputa. [file spectrum.01416-24-s0003.pdf]

Table S2. APT and control MGC and growth times for *Nocardia*, type and abundances of BF overgrowth in clinical sputa

Nocardia species	Strain	Replicate number	*Nocardia* MGC (CFU/ml)		*Nocardia* growth time (d)		Sputum number	BF type	BF overgrowth abundance	
			APT	Control	APT	Control			APT	Control
N. wallacei (n=7)	EML 1472	1	10^4	10^4	3	2	1	*Corynebacterium striatum*	Absence	Low
		2	10^3	10^3	3	2	2	*Serratia marcescens*	Low	Medium
	EML 1473	1	10^5	$>10^7$	9	>10	3	*Pseudomonas aeruginosa* + OPF	Low	High
		2	$>10^7$	$>10^7$	>10	>10	4	*Streptococcus pneumoniae*	High	High
	EML 1474	5	10^3	$>10^7$	3	>10	5	*Haemophilus influenzae* + OPF	Absence	High
		6	10^3	10^4	3	2	6	OPF	Absence	Low
		7	$>10^7$	$>10^7$	>10	>10	7	*Pseudomonas aeruginosa* + *Escherichia coli*	High	High
N. mexicana (n=5)	EML 1475	1	10^3	10^3	3	2	1	*Corynebacterium striatum*	Absence	Low
		2	10^4	10^5	2	2	6	OPF	Low	Low
	EML 1476	3	10^3	10^4	2	2	5	*Haemophilus influenzae* + OPF	Absence	Medium
		4	10^4	10^3	2	2	2	*Serratia marcescens*	Absence	Absence

		5	>10^7	>10^7	>10	>10	7	*Pseudomonas aeruginosa* + *Escherichia coli*	High	High
N. otitidiscaviarum (n=4)	EML 1477	1	10^3	>10^7	2	>10	3	*Pseudomonas aeruginosa* + OPF	Low	High
		2	10^3	>10^7	2	>10	7	*Pseudomonas aeruginosa* + *Escherichia coli*	Medium	High
	DSM 43242^T	3	10^5	10^3	2	2	2	*Serratia marcescens*	Absence	Absence
		4	>10^7	>10^7	>10	>10	8	*Stenotrophomonas maltophilia* + *Staphylococcus aureus + OPF*	High	High
N. farcinica (n=5)	EML 1478	1	10^4	>10^7	4	>10	3	*Pseudomonas aeruginosa* + OPF	Absence	High
		2	10^3	>10^7	3	>10	7	*Pseudomonas aeruginosa* + *Escherichia coli*	Medium	High
	EML 1479	3	>10^7	>10^7	>10	>10	5	*Haemophilus influenzae* + OPF	Absence	High
		4	10^6	>10^7	2	>10	4	*Streptococcus pneumoniae*	High	High
		5	10^6	10^5	5	5	2	*Serratia marcescens*	Medium	Medium

N. cyriacigeorgica (n=3)	EML 1480	1	10^6	$>10^7$	4	>10	3	*Pseudomonas aeruginosa* + OPF	Low	High
		2	10^3	10^3	3	2	2	*Serratia marcescens*	Absence	Absence
		3	10^3	10^4	3	2	6	OPF	Absence	Low
N. abscessus complex (n=7)	DSM 44432^T	1	$>10^7$	$>10^7$	>10	>10	3	*Pseudomonas aeruginosa* + OPF	Low	High
		2	$>10^7$	$>10^7$	>10	>10	8	*Stenotrophomonas maltophilia + Staphyloccocus aureus + OPF*	High	High
	EML 1481	1	$>10^7$	$>10^7$	>10	>10	3	*Pseudomonas aeruginosa* + OPF	Low	High
		2	$>10^7$	$>10^7$	>10	>10	1	*Corynebacterium striatum*	Low	Medium
		3	10^3	10^5	4	4	6	OPF	Absence	Absence
	EML 1482	1	$>10^7$	$>10^7$	>10	>10	3	*Pseudomonas aeruginosa* + OPF	Low	High
		2	$>10^7$	$>10^7$	>10	>10	4	*Streptococcus pneumoniae*	High	High

***N. nova complex* (n=4)**	EML 1483	1	10^6	$>10^7$	7	>10	3	*Pseudomonas aeruginosa* + OPF	Low	High
		2	$>10^7$	$>10^7$	>10	>10	4	*Streptococcus pneumoniae*	High	High
		3	$>10^7$	$>10^7$	>10	>10	3	*Pseudomonas aeruginosa* + OPF	Low	High
	DSM 44481^T	4	10^5	10^5	5	4	2	*Serratia marcescens*	Absence	High

CFU: Colony Forming Unit. APT: Amoebae Plate Test. BF: Bacterial Flora. OPF: Oropharyngeal Flora (e.g. *viridans Streptococci*, Coagulase negative *streptococci*, Gram negative bacilli, …).; Abundance of BF ranges from absence (0 colonies), low (<5 colonies), medium (6-50 colonies), high (> 50 colonies). EML: *Environnement Microbiologie Lyon* collection. DSM: *Deutsche Sammlung von Mikroorganismen collection*.
